# Strategies of adoptive T -cell transfer to treat refractory viral infections post allogeneic stem cell transplantation

**DOI:** 10.1186/s13045-019-0701-1

**Published:** 2019-02-06

**Authors:** Theresa Kaeuferle, Ramona Krauss, Franziska Blaeschke, Semjon Willier, Tobias Feuchtinger

**Affiliations:** 1 Department of Pediatric Hematology, Oncology, Hemostaseology and Stem Cell Transplantation, Dr. von Hauner University Children’s Hospital, Ludwig Maximilian University Munich, Lindwurmstrasse 4, 80337 Munich, Germany; 2 German Center for Infection Research (DZIF), Munich, Germany

**Keywords:** Adoptive T cell transfer, Virus-specific T cells, Refractory viral infections

## Abstract

**Background:**

Allogeneic hematopoietic stem cell transplantation (HSCT) can expose patients to a transient but marked immunosuppression, during which viral infections are an important cause of morbidity and mortality. Adoptive transfer of virus-specific T cells is an attractive approach to restore protective T -cell immunity in patients with refractory viral infections after allogeneic HSCT.

**Objectives:**

This narrative review summarizes clinical evidence and developments of almost 30 years of adoptive T -cell transfer. The review is based on evidence extracted from PubMed searches and the clinical and experimental work of the authors.

**Content:**

Viral infections after HSCT are frequently caused by the endogenous reactivation of persistent pathogens such as cytomegalovirus (CMV), Epstein-Barr virus (EBV), and adenovirus (AdV). Current antiviral medication is not satisfactory and does not treat the underlying pathophysiology which is the lack of specific T -cell immunity. Adoptive transfer of virus-specific T cells could be a potentially curative, pathogen-specific, and non-toxic treatment providing long-term immunity against the virus. The isolation of virus-specific T cells from a healthy donor and infusion into a recipient is known as adoptive T -cell transfer and has been performed in many patients using different treatment protocols. Based on basic research, new isolation protocols aim at a safe and fast availability of cellular products for adoptive T -cell transfer. We summarize preclinical and clinical data on each of the main pathogens and on the technical approaches currently available to target either single antigens or even multiple pathogens.

**Conclusion:**

Cellular therapy is considered as one of the major recent breakthroughs in medicine. Translation of this individualized treatment into first-line clinical routine is still limited. Main hurdles are availability of the technique, limited compatibility of classical phase III designs with cellular therapy, and regulatory restrictions. Multinational efforts are required to clarify the status of cellular treatment in first-line clinical routine with the overall objective to strengthen evidence-based treatment guidelines for the treatment of refractory viral infections *post* HSCT.

## Introduction

Allogeneic hematopoietic stem cell transplantation (HSCT) cures a variety of diseases, but it exposes patients to a transient severe immune deficiency. Since immune reconstitution after allogeneic HSCT can take 3 to 6 months [1–3], infections are a major cause of morbidity and mortality during this phase of immune deficiency. Taken together, infections cause 11% of all deaths after HSCT occurring with a median of 3 months after transplantation. About one third of infection-related deaths are caused by viruses, mainly human cytomegalovirus (CMV), Epstein-Barr virus (EBV), or human adenovirus (AdV) [4].

CMV is rarely associated with significant symptoms in healthy adults but causes severe complications during gestation and in immunocompromised patients [5]. EBV causes infectious mononucleosis but usually only mild, self-limiting disease followed by a lifelong latency of the virus in B cells. After HSCT, the latent virus can be reactivated and manifests as post-transplant lymphoproliferative disease (PTLD) [6, 7]. AdV is a widely spread virus, and the vast majority of pre-school children have had at least several respiratory or gastro-intestinal infections with AdV. AdV infections after HSCT show a particularly high incidence in pediatric patients. Local reactivations are often self-limiting, whereas systemic infections in the presence of a risk factor causing reduced T cell protection are associated with high morbidity and mortality [8].

**Fig. 1:**
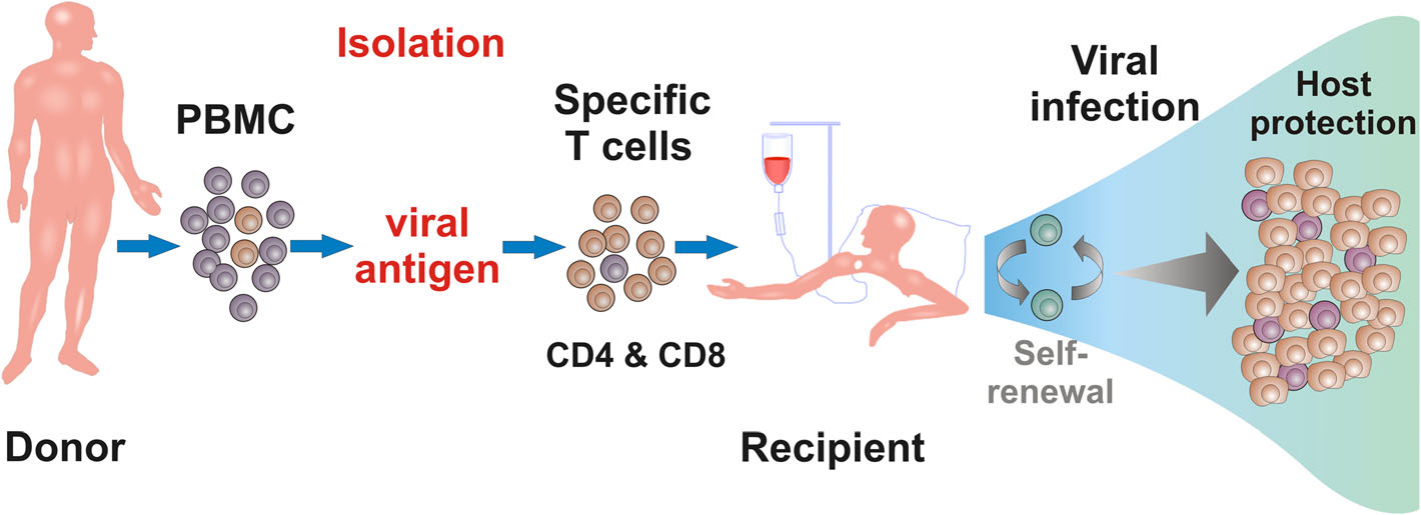
Adoptive T cell transfer. Adoptive transfer of multivirus-specific T cells from a healthy donor to a patient in order to treat refractory viral infections post stem cell transplantation (HSCT). Virus-specific T cells can be isolated by in vitro stimulation and expansion or direct selection of specific T cells ex vivo from peripheral blood of a seropositive donor

Conventional pharmacologic agents against AdV and EBV have limited efficacy and relevant toxicity [9, 10]. Pharmacological treatment of CMV shows better response rates, but toxicity and reactivation after treatment stop are frequent [11]. A sustained control of refractory viral infections will depend ultimately on the restoration of adequate antiviral immunity. Adoptive transfer of virus-specific T cells is an attractive approach to improve immune protection [7] (Fig. 1). This protection has been best described by the detection of specific T -cell responses in peripheral blood. For other predictive biomarkers, only few data exist. First, immunosuppression is likely to suppress antiviral T -cell responses. Under high doses of steroids, a success of adoptive T -cell therapy has not been possible. In theory, calcineurin inhibitors suppress T -cell responses, but systematic data are missing. Second, classical transplant parameters (donor type, human leukocyte antigen (HLA) match, underlying disease) have not been associated with differences in outcome of specific T -cell transfer. T cells from the initial HSCT-donor have been more successful compared to third-party donors [12]. Last but not the least, routine use of adoptive T -cell therapy requires T cells from seropositive donors. Therefore, we recommend testing of the donor for specific T -cell responses before adoptive T cell transfer.

Current development of chimeric antigen receptor (CAR) T cells for the treatment of leukemia and lymphoma has raised the question of a CAR T cell therapy against persistent viral infections *post* HSCT [13]. However, neither a combination of virus-specific T -cell receptor (TCR) with an anti-tumor CAR [14] nor an anti-viral CAR alone has been proven superior to an endogenous TCR.

In the context of recent reviews on this topic [15–17], this review illustrates the development of selection techniques for isolation of virus-specific T cells and summarizes almost 30 years of clinical evidence from studies using CMV-, EBV-, and AdV-specific T cells for adoptive T cell transfer.

## Development of selection techniques of virus-specific T cells

### Donor lymphocyte infusion

During the 1990s, viral infections after allogeneic HSCT frequently took a fatal course. The initial protocols of adoptive T -cell transfer were based on donor lymphocyte infusions (DLIs) which mediated antiviral activity with promising results [18, 19]. Unfortunately, unmanipulated DLIs provide relative high frequencies of alloreactive T cells resulting in a significant risk for graft-versus-host disease (GvHD) [20]. Therefore, different strategies have been developed to enrich, isolate, or purify virus-specific T cells.

### In vitro stimulation and expansion of virus-specific T cells

**Fig. 2:**
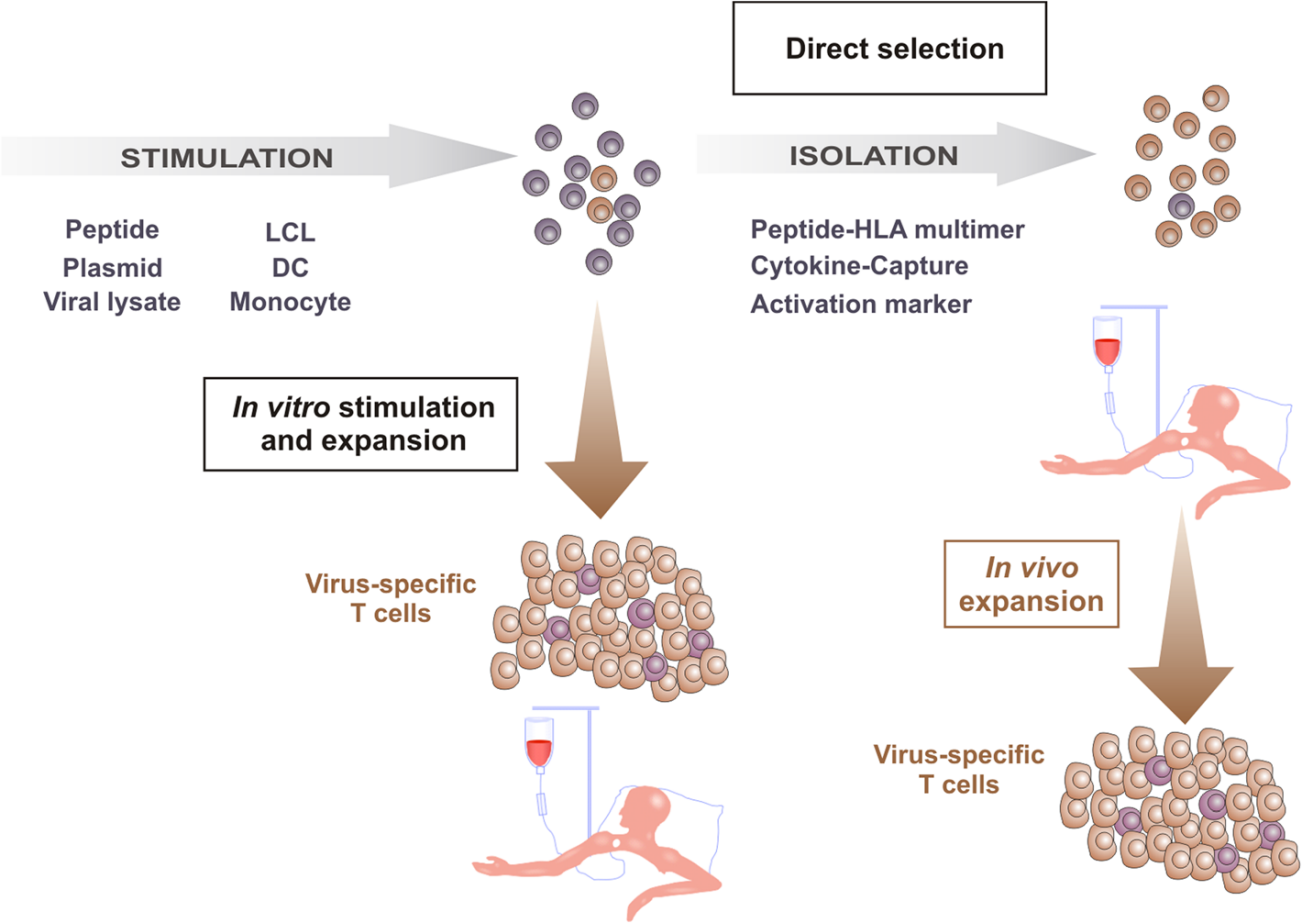
Selection techniques for the isolation of virus-specific T cells. Generation of virus-specific T cells by in vitro stimulation and expansion or direct selection. Firstly, cells are stimulated specifically via viral peptide/protein/lysate or antigen-presenting cells. Secondly, cells can either be used for in vitro expansion or isolation and direct infusion into the patient. Large amounts of virus-specific T cells can be obtained from a small starting volume of blood by in vitro stimulation and expansion. T -cell products from direct selection of virus-specific cells via peptide HLA multimers, cytokine-capture technique, or activation markers are obtained in small amounts and are infused into the patient where they expand under physiological conditions

Riddell and Greenberg set up a protocol in which solely virus-specific T cells are infused into the patient [21, 22]. They generated CMV-specific CD8^+^ T cells by ex vivo culture of donor peripheral blood mononuclear cells (PBMCs) in the presence of CMV-infected autologous fibroblasts followed by clonal expansion and depletion of CD4^+^ T cells. None of the treated patients showed significant side effects [21, 22]. However, these first results indicated the need of CD4^+^ T cells for prolonged survival of the adoptively transferred CD8^+^ T cell clones in vivo, so that Einsele and colleagues established a protocol for the isolation of CMV-specific polyclonal CD4^+^ and CD8^+^ T cells [23]. To remove potentially infective virus from the protocol, Peggs et al. pulsed autologous dendritic cells (DCs) with viral lysate instead of using CMV-infected autologous cells. Pulsed DCs were used as antigen-presenting cells (APCs) to restimulate CMV-specific T cells [24]. Rooney and colleagues generated EBV-specific T cells by successively stimulating donor-derived PBMCs with irradiated autologous EBV-transformed B cell lines (LCLs) to treat PTLD [25, 26] (Fig. 2).

Although virus-specific T -cell products can be generated in clinically useful numbers from a small volume of blood by in vitro stimulation and expansion, further efforts have been made to directly isolate virus-specific T cells from peripheral blood of a seropositive donor without in vitro expansion. Directly selected virus-specific T cells are supposed to proliferate more efficiently under physiological conditions in vivo than extensively in vitro cultured T cells. Moreover, it was shown that clonal expansion of virus-specific T cells in vitro is associated with an upregulated expression of the pro-apoptotic molecule Fas and a lack of CD28 expression, possibly due to overstimulation of the T cells [27].

### Direct selection of virus-specific T cells

For direct selection of virus-specific T cells, donor white blood cells are isolated ex vivo via peptide-HLA multimers, cytokine-capture method [28–30] after exposure to viral antigen, or methods based on expression and upregulation of activation molecules [31] on the surface of T cells. Virus-specific T cells are obtained in small amounts and are infused into the patient where they can expand effectively and induce viral clearance as well as sustained protection. However, this method implies a second blood donation of the HSCT donor, who additionally needs to show a sufficient population of virus-specific T cells to enable successful manufacturing of a T -cell product.

Human leukocyte antigen (HLA) multimers loaded with a virus-specific peptide allow highly specific labeling of virus-specific T cells [32, 33] (Fig. 2). Keenan et al. combined this labeling method with magnetic separation for purification of CMV-specific CD8^+^ T cells from PBMCs [34]. Recently, HLA multimers called streptamers were monomerized after isolation of peptide-specific T cells by adding a competitor followed by dissociation from the T cell before infusion into the patient [35]. However, since availability of multimers is restricted to HLA class I and specific epitopes, this technology enables generation of CD8^+^ T cells only against known immunodominant T -cell epitopes.

The cytokine-capture approach is a rapid method to isolate functional CD4^+^ and CD8^+^ T cells based on activation-induced release of interferon-γ (IFNγ) after stimulation with viral antigen (Fig. 2). Donor PBMCs are stimulated with viral antigen or viral lysate and labeled with a leukocyte-specific (CD45) antibody conjugated to an anti-IFNγ antibody. After the cytokine-capture period, specifically activated T cells are magnetically labeled via captured IFNγ on the surface allowing subsequent enrichment via a magnetic column [36, 37].

Another approach to isolate virus-specific T cells according to their activation after antigen stimulation is based on the activation-induced expression of specific surface molecules, such as CD137 (4-1BB) [38] and CD154 (CD40L) [39] and subsequent enrichment via a magnetic column.

### Generation of multivirus-specific T cells

Since the generation of adoptive T -cell therapy for each single virus in a separate manufacturing process is time- and cost-consuming, there are strong efforts to establish protocols for the generation of multivirus-specific T cells in one single step. One method is based on the generation of EBV-specific T cells by repetitively stimulating and expanding PBMCs with LCLs [25, 26]. Leen et al. used irradiated autologous LCLs transduced with an adenoviral vector expressing the CMV antigen pp65. These LCLs served as antigen-presenting cells for the inherent EBV antigens, the adenoviral hexon antigen from the capsid of the vector, and pp65 from the vector transfection to stimulate PBMCs specific for CMV, EBV, and AdV [40]. These manufacturing processes require several weeks.

Therefore, several efforts were made to reduce the manufacturing time like stimulation of donor-derived PBMCs with autologous monocytes transduced with either Ad5f35-CMV-pp65 (AdV and CMV) vector or Ad5f35-EBV-LMP2 (AdV and EBV) vector. After stimulation of CMV-, EBV-, and AdV-specific T cells, an isolation step could be added using the cytokine-capture technique [41]. Another approach was the use of DCs nucleofected with plasmids encoding pp65 (CMV), IE1 (CMV), LMP2 (EBV), EBNA1 (EBV), BZLF1 (EBV), Hexon (AdV), and Penton (AdV). After nucleofection, the DCs were used as APCs for stimulation of CMV-, EBV-, and AdV-specific T cells. Generation of multivirus-specific T cells with this protocol can be performed in 11 to 12 days [42].

Furthermore, multivirus-specific T cells have already been produced using direct isolation via the cytokine-capture technique [43], and Khanna et al. described a protocol where multipathogen-specific T cells expressing CD154 were directly isolated via magnetic cell separation [31]. A comparison of multivirus-specific T cells isolated based on either CD137 expression or IFNγ production showed no significant differences in functionality or CD4^+^/CD8^+^ T -cell frequencies [44].

## Clinical evidence

### Cytomegalovirus

The first clinical application of adoptive T -cell therapy against viral infections was done with CMV-specific T cells in 1992 [22] (Table 1). Riddell and Greenberg treated three patients with repetitive infusions of escalating numbers of in vitro expanded CMV-specific CD8^+^ T -cell clones over 4 weeks. None of the three patients showed severe side effects and also none of them developed CMV viremia or pneumonia [22]. Accordingly, a further study with 14 patients using in vitro expansion to generate CMV-specific T cells confirmed the absence of toxic effects and confirmed CMV-specific immune reconstitution in all treated patients [45]. Einsele and colleagues extended the protocol and treated eight infected patients with polyclonal CMV-specific CD4^+^ and CD8^+^ T cells. Five out of seven evaluable patients cleared the infection completely, whereas one of these patients did not respond until a second infusion of CMV-specific T cells [23]. Another study performed by Peggs et al. showed massive expansion of infused polyclonal CMV-specific CD4^+^ and CD8^+^ T cells after adoptive transfer and recovery of immune response in all patients [46]. The first adoptive transfer of tetramer-selected CD8^+^ T cells was done by Cobbold and his colleagues. Despite the relatively low doses in comparison to the other studies, eight of nine patients cleared the virus completely [47]. Two patients treated with CMV-specific CD8^+^ T cells generated via the reversible streptamer technique showed long-lasting responses and control of CMV viremia [48]. In a recent phase I/IIa study, patients were treated with streptamer-isolated CMV-specific T cells, generated either from their stem cell donor or from partially HLA-matched third-party donors [12]. Seven of seven eligible patients treated with stem cell donor-derived T cells reduced or cleared viral load whereas five of eight patients responded after transfer of third-party donor-derived T cells [12]. Adoptive T -cell transfer of CMV-specific T cells isolated by cytokine-secretion assay led to viral clearance or significant reduction of viral load in 15 of 18 treated patients [28].

**Table 1:** Clinical evidence for adoptive transfer of CMV-specific T cells

Reference	Method	No. of patients	Results	Dose
In vitro stimulation and expansion				
Riddell et al. (1992) [22]	Allogeneic CMV-spec. CD8^+^ clones	3	3/3 prevention of viremia and pneumonia	3.3 × 10^7^–10^9^ cells/m^2^
Walter et al. (1995) [45]	Allogeneic CMV-spec. CD8^+^ clones phase I	14 (11)	11/11 prevention of CMV infection	3.3 × 10^7^–10^9^ cells/m^2^
Einsele et al. (2002) [23]	Allogeneic CMV-spec. polyclonal CD8^+^ and CD4^+^ T cells	8	5/7 evaluable patients eliminated infection	10^7^ cells/m^2^
Peggs et al. (2003) [46]	Allogeneic CMV-specific polyclonal CD8^+^ and CD4^+^ T cells	16	14/16 no viral reactivation, reconstitution of antiviral immunity	10^5^ cells/kg
Perruccio et al. (2005) [56]	Allogeneic CMV-specific CD4^+^ clones	25 prophylaxis	7/25 patients had CMV-reactivation, 5/25 patients developed CMV-disease (3 eliminated infection)	10^5^–3 × 10^6^ cells/kg
Meji et al. (2012) [57]	CMV-specific polyclonal CD8^+^ and CD4^+^ T cells phase I/II	6	6/6 patients eliminated infection	0.9 × 10^4^–3.1 × 10^5^ cells/kg
Pei et al. (2017) [58]	CMV-specific cytokine induce effector cells phase I	32	27/32 responded	0.66–15.41 × 10^7^ CD8^+^ and 0.68–9.25 × 10^5^ CD4^+^
Withers et al. (2017 and 2018) [55, 59]	CMV-specific third-party CD8+ and CD4+ T cells phase I	27	26/27 responded	1.37–5.0 × 10^7^ cells/m^2^
Direct isolation via peptide-HLA multimers				
Cobbold et al. (2005) [47]	Allogeneic CMV-specific CD8^+^ T cells using MHC-I-tetramers	9	8/9 patients eliminated infection	1.2–33 × 10^3^ cells/kg
Schmitt et al. (2011) [48]	Allogeneic CMV-specific CD8^+^ T cells using MHC-I-streptamers	2	2/2 control of CMV-viremia	0.37 and 2.2 × 10^5^ cells/kg
Uhlin et al. (2012) [60]	Allogeneic CMV-specific CD8^+^ T cells using MHC-I-pentamers	5	4/5 responders	0.8–24.6 × 10^4^ cells/kg
Blyth et al. (2013) [61]	Allogeneic CMV-specific polyclonal CD8^+^ and CD4^+^ T cells phase II	50 Prophylaxis	41/50 did not require CMV-directed pharmacotherapy	2 × 10^7^ cells/m^2^
Neuenhahn et al. (2017) [12]	Allogeneic CMV-specific CD8^+^ T cells using MHC-I-streptamers phase I/IIa	16	Stem cell donor-derived: 7/7 responders third-party transfer: 5/8 responders	6.3 × 10^6^ cells (HSCT donor) 1.4 × 10^7^ cells (third-party donor)
Direct isolation via cytokine-capture technique				
Feuchtingeret al. (2010) [28]	CMV-specific polyclonal CD8^+^ and CD4^+^ T cells	18	15/18 responders	1.2–166 × 10^3^ cells/kg
Peggs et al. (2011) [62]	CMV-specific polyclonal CD8^+^ and CD4^+^ T cells phase I/II	18	Prophylaxis: 6/7 virus-free Pre-emptive: 2/11 required no antiviral drug treatment	Median: 3.5 × 10^4^ cells/kg
Kàllay et al. (2018) [43]	CMV-specific polyclonal CD8^+^ and CD4^+^ T cells	3	2/3 viral clearance 1/3 decrease in viral load	7.5–16.2 × 10^4^ cells/kg

### Epstein-Barr virus

Rooney et al. published a first study with EBV-specific T cells generated by repetitive stimulation of donor-derived PBMCs with irradiated autologous EBV-transformed B cell lines for the treatment of EBV-associated PTLD [25] (Table 2). Ten patients received adoptive T cell therapy who either had EBV reactivation or were treated prophylactically. In the three patients with EBV reactivation, EBV DNA decreased to normal. None of the seven patients who received EBV-specific T cells as prophylaxis developed EBV disease [25]. This approach was further established in a later study with 39 patients who received prophylactic infusion of EBV-specific T cells. None of the 39 patients developed EBV-related PTLD in comparison to 11.5% in a control group of 61 patients who did not receive EBV-specific adoptive T cell therapy [26]. With genetically marked adoptively transferred EBV-specific T cells, it could be shown that donor-derived T cells were present in the patient even 105 months after adoptive T cell transfer [49]. Haque and colleagues treated eight patients with progressive PTLD in a pilot study with EBV-specific polyclonal CD8^+^ and CD4^+^ T cells generated from partially HLA-matched unrelated donors [50]. Four of the eight patients had a complete or partial remission, and none of the patients developed GvHD [50]. In a following multicenter phase II clinical trial, the response rate after 6 months was 52% [51]. Notably, in this study, infusion of higher amounts of CD4^+^ T cells led to a significantly better outcome. These data suggest that infusion of EBV-specific T cells induce relatively lower response rates compared to that of CMV-specific T cells. A possible explanation is that CMV is mainly controlled by a single antigen pp65 with an extraordinary high immunogenicity, whereas EBV antigens induce less strong T cell responses. Nevertheless, by using the same technique for different viruses, a comparable result could be achieved: Icheva et al. were able to treat 10 patients with PTLD- and EBV-related complications with small doses of EBNA1-specific T cells isolated by cytokine-capture technique and describe a clinical and virological response in 7 out of 10 patients [29].

**Table 2:** Clinical evidence for adoptive transfer of EBV-specific T cells

Reference	Method	No. of patients	Results	Dose
In vitro stimulation and expansion				
Rooney et al. (1995) [25]	Allogeneic EBV-specific CD8^+^ T cells	10	Therapy: 3/3 responders Prophylaxis: 7/7 virus free	0.2–1.2 × 10^8^ cells/m^2^
Rooney et al. (1998) [26]	Allogeneic EBV-specific CD8^+^ T cells	39	Prophylaxis: all PTLD free	0.2–1.0 × 10^8^ cells/m^2^
Haque et al. (2002) [50]	Allogeneic EBV-specific polyclonal CD8^+^ and CD4^+^ T cells phase I/II	8	4/8 remission	10^6^ cells/kg
Haque et al. (2007) [51]	Allogeneic EBV-specific polyclonal CD8^+^ and CD4^+^ T cells phase II	33	14/33 complete remission 3/33 partial response	2 × 10^6^ cells/kg
Heslop et al. (2010) [49]	Allogeneic EBV-specific CD8^+^ T cells	114	Therapy: 11/13 complete response prophylaxis: all PTLD free	1–5 × 10^7^ cells/m^2^
Doubrovina et al. (2012) [63]	Allogeneic EBV-specific CD8^+^ T cells	19	13/19 complete response	10^6^ cells/kg
Gallot et al. (2014) [64]	Allogeneic EBV-specific polyclonal CD8^+^ and CD4^+^ T cells phase I/II	11	4/10 responders	5 × 10^6^ cells/kg
Withers et al. (2017 and 2018) [55, 59]	EBV-specific third-party CD8+ and CD4+ T cells phase I	1	0/1 responded	1 infusion of 1.37–5.0 × 10^7^ cells/m^2^
Direct isolation via peptide-HLA multimers				
Uhlin et al. (2010) [65]	Allogeneic EBV-specific CD8^+^ T cells using MHC-I-pentamers	1	1/1 complete response	1.1 × 10^4^ cells/kg and 2 × 10^4^ cells/kg
Direct isolation via cytokine-capture technique				
Moosmann et al. (2010) [66]	EBV-specific polyclonal CD8^+^ and CD4^+^ T cells	6	3/6 responders	0.4–9.7 × 10^4^ cells/kg
Icheva et al. (2013) [29]	EBV-specific polyclonal CD8^+^ and CD4^+^ T cells	10	7/10 responders	0.15–53.8 × 10^3^ cells/kg
Kàllay et al. (2018) [43]	EBV-specific polyclonal CD8^+^ and CD4^+^ T cells	2	2/2 responders	1.8–2.3 × 10^4^ cells/kg

### Adenovirus

In a first study using adoptive transfer of AdV-specific T cells, nine children with systemic AdV infection after allogeneic HSCT were treated with cells isolated by cytokine-capture technique based on IFNγ secretion [52] (Table 3). Despite the relatively low number of infused T cells, five of six evaluable patients cleared the infection or showed a decrease in viral load which was associated with expansion of AdV-specific T cells in vivo. These results indicate that even low numbers of adoptively transferred virus-specific T cells are able to expand in vivo in the presence of viral antigen. Further, no acute toxicities or GvHD induction have been documented [52]. Virus-specific T cells generated by in vitro stimulation and expansion were administered to two patients with rising AdV load and led to complete clearance or more than 1.5 log reduction of viral load in all of the three patients [53]. Refractory AdV infection was treated with hexon-specific T cells generated via cytokine-capture technique in a study with 30 patients. Twenty-one of these patients responded to adoptive T cell transfer without relevant side effects [30].

**Table 3:** Clinical evidence for adoptive transfer of AdV-specific T cells

Reference	Method	No. of patients	Results	Dose
In vitro stimulation and expansion				
Geyeregger et al. (2014) [53]	Allogeneic AdV-specific polyclonal CD8^+^ and CD4^+^ T cells	2	1/2 complete response 1/2 partial response	10^4^ CD3^+^ cells/kg
Withers et al. (2017 and 2018) [55, 59]	AdV-specific third-party CD8+ and CD4+ T cells phase I	1	1/1 responded	1 infusion of 1.37–5 × 10^7^ cells/m^2^
Direct isolation via peptide-HLA multimers				
Uhlin et al. (2012) [60]	Allogeneic AdV-specific CD8^+^ T cells using MHC-I-pentamers	8	5/6 responders	3.1 × 10^4^ and 1.7 × 10^4^ cells/kg
Direct isolation via cytokine-capture technique				
Feuchtinger et al. (2006) [52]	AdV-specific polyclonal CD8^+^ and CD4^+^ T cells	9	5/6 responders	1.2–50 × 10^3^ cells/kg
Qasim et al. (2013) [67]	AdV-specific polyclonal CD8^+^ and CD4^+^ T cells	5	3/5 responders	10^4^ cells/kg
Feucht et al. (2015) [30]	AdV-specific polyclonal CD8^+^ and CD4^+^ T cells	30	21/30 responders	0.3–24 × 10^3^ CD3^+^ cells/kg
Kàllay et al. (2018) [43]	AdV-specific polyclonal CD8^+^ and CD4^+^ T cells	1	1/1 responder	2.7 × 10^4^ cells/kg

### Multivirus-specific (CMV, EBV, and AdV) T cell therapy

The first clinical application of multivirus-specific T cells was published in 2006 by Leen and her colleagues [40] (Table 4). Adoptive transfer was performed in 11 patients where trivirus-specific T cells, generated by in vitro stimulation and expansion, expanded and provided long-term immunity. Notably, all patients with a CMV, EBV, or AdV infection cleared the infection. In contrast to CMV- and EBV-specific T cells, the expansion of AdV-specific T cells seemed to depend on the presence of adenoviral antigen: CMV- and EBV-specific cytotoxic T lymphocytes (CTLs) consistently expanded in vivo after administration, whereas adenovirus-specific CTLs expanded only in individuals with active or recent adenoviral infection. In 2009, Leen et al. generated bivirus-specific T cell lines by in vitro stimulation and expansion [54]. AdV- and EBV-specific T cells were administered to pediatric transplantation recipients with partially HLA-matched and haploidentical stem cell grafts. None of the patients developed GvHD and none of these 13 high-risk recipients developed EBV-associated lymphoproliferative disease, while two of the subjects showed resolution of their adenoviral disease [54]. In a recent study, Kállay et al. treated three patients with multivirus-specific T cells generated by direct isolation via the cytokine-capture technique [43]. All patients became asymptomatic and decreased/cleared viral load, but one patient died later due to invasive aspergillosis.

**Table 4:** Clinical evidence for adoptive transfer of multivirus-specific T cells

Reference	Method	No. of patients	Results	Dose
In vitro stimulation and expansion				
Leen et al. (2006) [40]	Allogeneic CMV-, EBV, and AdV-specific CD8^+^ T cells	11	All patients eliminated the viral pathogen	5 × 10^6^–1 × 10^8^ cells/m^2^
Leen et al. (2009) [54]	Allogeneic EBV- and AdV-specific CD8^+^ T cells	13	Therapy: 2/2 AdV clearance prophylaxis: 13/13 no PTLD	0.5–13.5 × 10^7^ cells/m^2^
Gerdemann et al. (2013) [68]	Allogeneic CMV-, EBV, and AdV- specific CD8^+^ T cells phase I/II	10 (infections: 3 CMV, 1 AdV, 2 EBV, 2 EBV + AdV, 2 CMV + AdV)	8/10 complete responses	5 × 10^6^–2 × 10^7^ cells/m^2^
Withers et al. (2017 and 2018) [55, 59]	Third-party CD8+ and CD4+ CMV-, EBV, AdV, and varicella-zoster virus-specific T cells phase I	1	1/1 responded	3 infusions of 1.37–5 × 10^7^ cells/m^2^
Direct isolation via cytokine-capture technique				
Kàllay et al. (2018) [43]	CMV- and EBV-specific or CMV- and AdV-specific CD8^+^ and CD4^+^ T cells	3 (infections: 2 CMV + AdV, 1 CMV + EBV)	3/3 responders	3.2–4.8 × 10^4^ cells/kg

## Conclusion and future perspective

Viral infections refractory to antiviral chemotherapy are a life-threatening condition in immunocompromised hosts. Clinical trials using CMV-, EBV-, and AdV-specific T cells for adoptive T -cell transfer have shown that T -cell therapy is an attractive approach to restore protective antiviral T -cell immunity. In almost 30 years of adoptive T -cell transfer, 74% of 246 evaluable, published patients responded to the treatment. In total, 85% responded to CMV-specific T cell transfer, 62% to EBV-specific T cells, and 74% to AdV-specific T cell transfer.

Dosing of virus-specific T cells depends on the risk of GvHD, method of generation, and the grade of HLA match/mismatch. For ex vivo-generated T cells, we currently recommend an upper dose limit of 2.5 × 10^4^/kg recipient body weight CD3^+^ cells in HLA-mismatched/haploidentical donors and 1 × 10^5^/kg in HLA-matched donors. A recommendation of a lower threshold does not exist at the moment. Lowest successful doses have been published as low as a few hundred cells. Protocols with longer in vitro culture steps have used higher doses.

Developing techniques for manufacturing virus-specific T -cell products has overcome initial difficulties of adoptive T -cell transfer. Nevertheless, the regulatory hurdles, logistics, and time-consuming selection techniques for producing virus-specific T -cell grafts have limited widespread application of this therapy. Off-the-shelf production of a T -cell product is promising, but clinical efficacy has not yet been fully confirmed in placebo-controlled studies. Third-party T -cells showed clinical benefits [55], but clarification of persistence in vivo remains to be investigated. A current multinational, placebo-controlled, phase III clinical trial (TRACE) aims to generate clinical evidence data to allow the inclusion of adoptive transfer of virus-specific T cells into evidence-based treatment guidelines and make it available as standard treatment for refractory viral infections post HSCT in the future.

## Abbreviations

AdV: Adenovirus
APCs: Antigen-presenting cells
CMV: Cytomegalovirus
DCs: Dendritic cells
DLI: Donor lymphocyte infusion
EBV: Epstein-Barr virus
GvHD: Graft-versus-host disease
HLA: Human leukocyte antigen
HSCT: Hematopoietic stem cell transplantation
IFNγ: Interferon-γ
LCLs: Autologous EBV-transformed B cell lines
PBMC: Peripheral-blood mononuclear cells
PTLD: Post-transplant lymphoproliferative disease
TRACE: Multivirus-specific T cell transfer post SCT vs AdV, CMV, and EBV infections
